# Antiganglioside Antibodies and Inflammatory Response in Cutaneous Melanoma

**DOI:** 10.1155/2020/2491265

**Published:** 2020-08-13

**Authors:** Corina Daniela Ene, Mircea Tampa, Ilinca Nicolae, Cristina Iulia Mitran, Madalina Irina Mitran, Clara Matei, Ana Caruntu, Constantin Caruntu, Simona Roxana Georgescu

**Affiliations:** ^1^“Carol Davila” Nephrology Hospital, 4 Calea Grivitei, 010731 Bucharest, Romania; ^2^“Carol Davila” University of Medicine and Pharmacy, 37 Dionisie Lupu, 020021 Bucharest, Romania; ^3^“Victor Babes” Clinical Hospital for Infectious Diseases, 281 Mihai Bravu, 030303 Bucharest, Romania; ^4^Department of Oral and Maxillofacial Surgery, “Carol Davila” Central Military Emergency Hospital, 134 Calea Plevnei, 010825 Bucharest, Romania; ^5^Faculty of Medicine, “Titu Maiorescu” University, 22 Dambrovnicului, 031593 Bucharest, Romania; ^6^“Prof. N. Paulescu” National Institute of Diabetes, Nutrition and Metabolic Diseases, 22-24 Gr. Manolescu, Bucharest 011233, Romania

## Abstract

**Introduction:**

Endogenously produced antiganglioside antibodies could affect the evolution of cutaneous melanoma. Epidemiological and experimental evidence suggest “chronic inflammation” to be one of the hallmarks in skin cancers. The aim of the study was to characterize the relation between antiganglioside antibodies and inflammation in cutaneous melanoma focusing on gangliosides GM1, GM2, GM3, GD1a, GD1b, GT1b, GQ1b. *Material and Method*. We performed an observational study that included 380 subjects subdivided into three groups: patients with metastatic melanoma (170 cases), patients with primary melanoma (160 cases), and healthy subjects (50 subjects). The assessment of antiganglioside antibodies, IgG, and IgM classes, against -GM1, -GM2, -GM3, -GD1a, -GD1b, -GT1b, -GQ1b was performed using immunoblot technique (EUROLine kit).

**Results:**

The presence of IgG and IgM antiganglioside antibodies in primary melanoma was (%), as follows: anti-GM1 (5.0 and 13.1), -GM2 (1.8 and 18.1), -GM3 (0.6 and 5.6), -GD1a (0.6 and 15.0), -GD1b (3.7 and 10.7), -GT1b (0.0 and 13.1), -GQ1b (0.0 and 5.0). In metastatic melanoma, the level of antiganglioside antibodies was significantly lower compared with primary melanoma (*p* < 0.05), while in the control group they were absent. Antiganglioside antibodies anti-GM1 and -GD1a were positively correlated, while anti-GM3, -GD1b, and -GT1b were negatively associated with the inflammatory markers, interleukin 8 (IL-8), and C reactive protein (CRP).

**Conclusions:**

Tumour ganglioside antigens generate an immune response in patients with primary melanomas. The host's ability to elaborate an early antiganglioside response could be considered as a defence mechanism, directed toward eliminating a danger signal from the tumour microenvironment. Antiganglioside antibodies associated with inflammation markers could be used as diagnostic, monitoring, and treatment tools in patients with cutaneous melanoma.

## 1. Introduction

Gangliosides are a group of bioactive glycolipids, located on the outer face of cell membranes. These glycolipids play a major role in cell proliferation, differentiation, migration, apoptosis, signal transduction, cell adhesion, modulating growth factor or hormone receptor, antigen recognition, protein trafficking, viral transformation, and oncogenesis [1–5]. Atypical expression of some ganglioside antigens associated with certain tumours (neuroblastomas, melanomas, gliomas, lymphomas, small cell lung cancer, and prostate cancer) and furthermore could play an important role in cancer immunotherapy [6–8]. Gangliosides that are released in extracellular spaces could have dual action, antitumor, and protumour effect [8–11]. Data regarding the endogenous immune response directed toward tumour gangliosides and the significance of this response are limited. A series of studies performed in in vivo experimental models and in vitro in murine and human cancer cells have shown that monoclonal antiganglioside antibodies have antitumor potential. These antibodies exert numerous antitumor effects through various mechanisms. An important mechanism is the translocation of gangliosides from the plasmatic membrane into the intracellular spaces, so, binding of antibodies to the surface of the tumor cells and complement activation that leads to cell lysis mediated by complement-dependent cytotoxicity and antibody-mediated cellular cytotoxicity [4, 7, 8]. Antiganglioside antibodies modulate ceramide synthesis [7, 10, 12]; reception and transduction of the cytotoxic signal [7]; they are involved in suppression or induction of cell death through different pathways (apoptosis, necrosis, oncogenes-like, structural, and functional changes of mitochondria, accumulation of reactive oxygen species, acetylation of gangliosides, accumulation of sphingosine, sphingamine, ceramides) [2, 10, 12]. Proteomic studies showed that antiganglioside antibodies could induce changes like the disruption of signalling systems (P38-MAPK, PARP, JNK1/2/3, METc, ERK1/2, P13K/AKT, and FAK), modulation of the level, and function of transcription factors (P53, SP1, MYCN, and HSF1), regulating the balance between apoptosis-inducing and apoptosis-suppressing factors (cysteine-aspartyl-proteases, Bax, Bcl-2) [2, 7, 12–14]. These antibodies stimulate the cytotoxicity of chemotherapeutic drugs and small molecule inhibitors [2, 7]. As a result, antiganglioside antibodies could be used as diagnostic, monitoring, and treatment tools in cancer patients [4, 8].

Ganglioside levels are increased in malignant melanocytes and represent an important topic of research [15, 16]. Several researchers have emphasized the role of glycolipids as markers of melanoma. A study analysing the expression of gangliosides in melanocyte lines and melanoma cell lines found out an increased expression of GD3 synthase genes in melanoma cells but not in melanocytes. The same results were obtained for GM2/GD2 synthase [15]. It seems that gangliosides induce cell proliferation and invasion through p130Cas and paxillin in melanoma cells [17].

Inflammatory mechanisms play an important role in melanoma. Multiple studies have shown that plasma levels of C reactive protein (CRP) increase during tumor proliferation and several relations have been evaluated, CRP-survival relationship, CRP-response therapy, CRP-inflammation. Nowadays, CRP is considered a true marker for assessing inflammation in melanoma, as well as a marker for response to treatment. Prospective studies have provided consistent results in the predictive value of CRP in neoplastic disease proving high sensitivity and specificity [14]. In addition, in melanoma elevated levels of CRP may reflect the amount and activity of circulating proinflammatory cytokines, e.g., interleukin 8 (IL-8). IL-8 plays a crucial role in regulating cell function for host defence and for developing natural immunity [13, 18]. Moreover, IL-8 is released by various cell types, including polymorphonuclear neutrophils (PMNs), monocytes, T lymphocytes, and endothelial cells, upon exposure to inflammatory stimuli. Melanoma cells have been reported to express IL-8 and this influences their oncogenic properties [12, 19]. IL-8 follows the evolution of melanoma, progression, and regression under treatment, reflecting the stage of the disease [20–23].

Based on these accumulating data, we have investigated antiganglioside antibodies in correlation with other inflammatory markers (IL-8, CRP) and the clinical evolution of the melanoma patients. Clarifying these relations could significantly improve the prediction of clinical outcomes. Furthermore, it can lead to the development of appropriate therapeutic strategies in patients with cutaneous melanoma.

## 2. Material and Method

### 2.1. Patients

We performed an observational, prospective study during 7 years in Clinical Hospital for Infectious and Tropical Diseases “Victor Babes”—Dermatology Department, Bucharest. The study was approved by the Ethics Committee of the Hospital. All participants agreed to be included in research studies without prejudice of the diagnosis or personal image, and signed the informed consent according to the Declaration of Helsinki.

The study included adult patients with cutaneous melanoma, with no other pathologies and no treatment for the primary disease. Exclusion criteria were age under 18 years, pregnancy, alcohol use, melanoma under treatment.

We performed an observational study that included 380 subjects subdivided in three groups: patients with metastatic melanoma (170 cases), patients with primary melanoma (160 cases), and healthy subjects with matching sex and age (50 subjects). Patients were selected and examined according to 2019 ESMO Clinical Practice Guidelines for melanoma diagnosis, based on clinical, histopathological, immunohistochemical, and imagistic data. All the events related to the progression of the disease were recorded (relapse, metastasis, neurotoxicity, hyper reactivation of the immune system upon treatment). The group characteristics were similar for age and sex: the primary melanoma group included 83 women and 77 men with a mean age of 45.7 ± 12.3 years, the metastatic melanoma group included 88 women and 82 men with a mean age of 51.3 ± 13.7 years, and the control group included 27 women and 23 men with mean age of 43.1 ± 10.8 years.

### 2.2. Materials and Reagents

**Figure 1:**
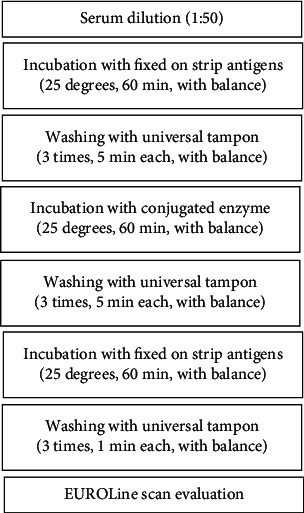
Antiganglioside detection.

In this work, the assessment of antiganglioside antibodies was made by the immunoblot technique, using EUROLine kits (Figure 1). This method allows the evaluation of antibodies, IgG, and IgM classes, against GM1, GM2, GM3, GD1a, GD1b, GT1b, and GQ1b from serum/plasma. The kit contains strips marked with purified antigens (Table 1).

The evaluation of antiganglioside antibodies was performed using the EUROLine Scan software. After reading the signal intensity on the strips marked with ganglioside antigens, the results were evaluated and the results are presented as optical sensibility. The assessment of IL-8 was performed by the ELISA method using Enzo Life Science reagents with TECAN analyser, and the results are presented as pg/dl. CRP was assessed by immunoturbidimetry using Human reagents and HumaStar300 analyser; the results are presented as mg/dl.

### 2.3. Statistical Analysis

All the results were analysed using IBM SPSS Statistics 2015. We evaluated the normality of data distribution using the Kolmogorov-Smirnov test. The variation between groups was determined using the parametric tests-*t* test, when two groups were compared, or ANOVA test, when more groups were compared, and nonparametric tests like Mann-Whitney or Wilcoxon. The correlation between groups was evaluated using linear regression and Pearson coefficient. *p* < 0.05 was considered with statistical significance.

## 3. Results

Anti-GM1, -GM2, -GM3, -GD1a, -GD1b, -GT1b, and -GQ1b autoantibodies determined in primary, metastatic melanoma had different serological profiles compared to the control group. The presence of IgG and IgM antiganglioside antibodies in primary melanoma was (%), as follows: antiGM1 (5.0 and 13.1), antiGM2 (1.8 and 18.1), antiGM3 (0.6 and 5.6), antiGD1a (0.6 and 15.0), antiGD1b (3.7 and 10.7), antiGT1b (0.0 and 13.1), antiGQ1b (0.0 and 5.0). In metastatic melanoma, IgG and IgM antiganglioside antibodies had the following profile (%): anti-GM1 (1.2 and 2.9), anti-GM2 (0 and 2.3), anti-GM3 (0 and 0.2), anti-GD1a (0 and 1.7), anti-GD1b (0 and 2.9), anti-GT1b (0 and 0.6), and anti-GQ1b (0 and 0.6). In the control group, antiganglioside autoantibodies were absent.

**Figure 2:**
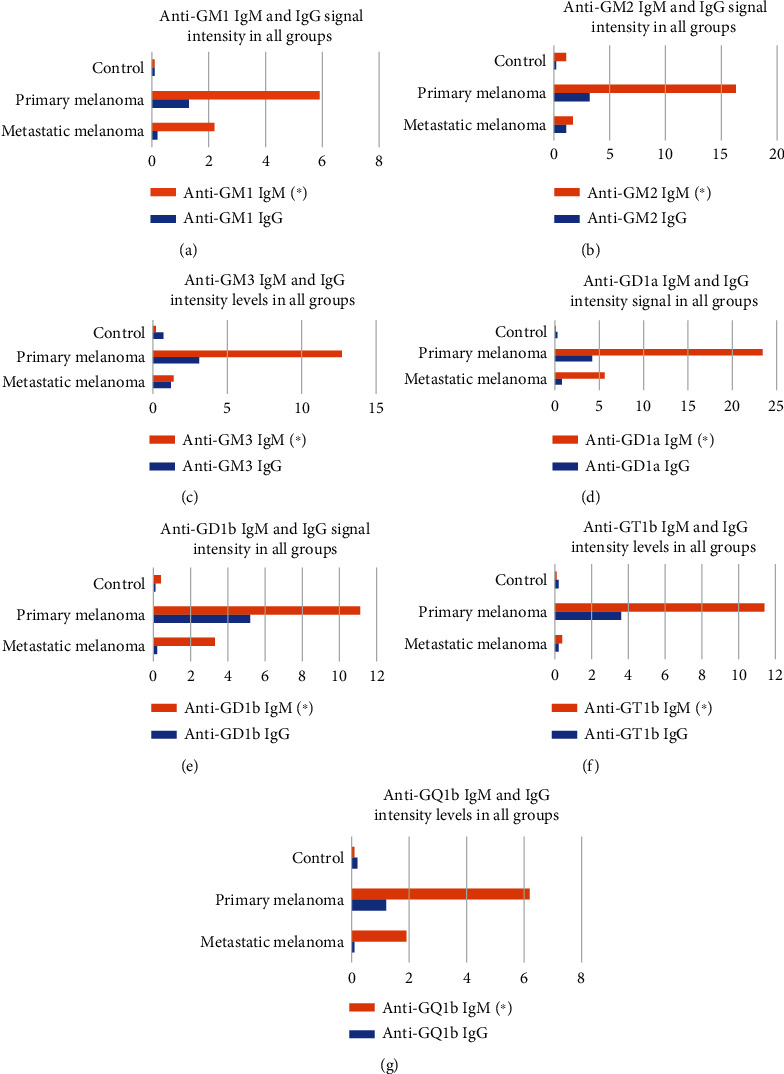
Antiganglioside signal intensity in all groups. ^∗^*p* < 0.05.

The assessment for IgG anti-GM1, anti-GM2, anti-GM3, anti-GD1a, anti-GD1b, anti-GT1a, and anti-GT1b showed extremely low signal intensity in all groups (Figure 2). When comparing the mean of signal intensity for IgG, no statistical differences were observed between groups. We obtained a statistically significant difference in IgM anti-GM1, anti-GM2, anti-GM3, anti-GD1a, anti-GD1b, anti-GT1a, and anti-GT1b when comparing primary melanoma, respectively, metastatic melanoma to the control group, and once more when comparing primary versus metastatic melanoma (Table 2).

**Figure 3:**
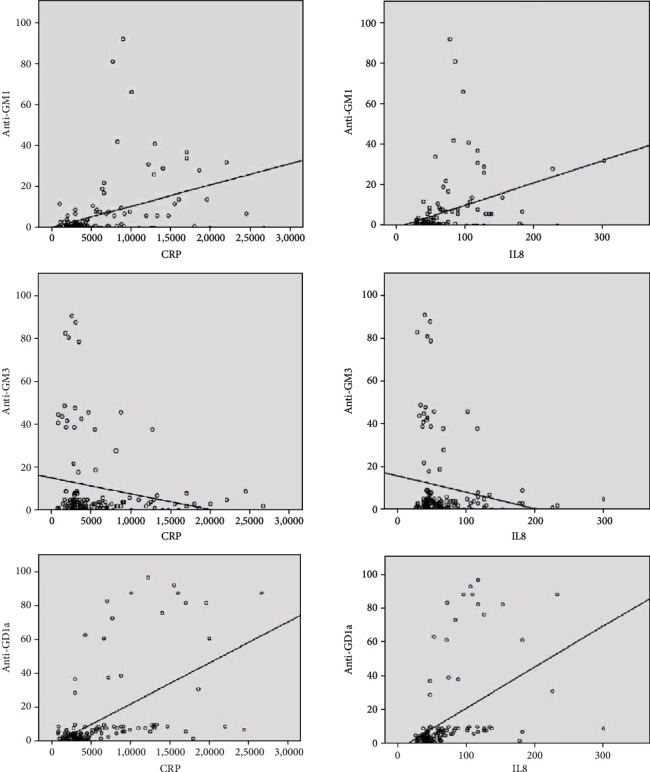
Anti-GM1, anti-GM3, and anti-GD1a in relation to CRP and IL-8 in the primary melanoma group.

To evaluate if the presence of IgM antibodies was associated with melanoma development, we determined their relation to inflammatory factors (IL-8 and CRP) recommended by AJCC for melanoma staging (Table 3). IL-8 levels were statistically significantly increased in primary melanoma (68.9 ± 17.2 pg/ml) and in metastatic melanoma (74.2 ± 24.2 pg/ml) when compared with the control group (10.9 ± 4.6 pg/ml). CRP levels were found in primary (1.07 ± 0.88 ng/ml) and metastatic melanoma (1.85 ± 0.62 ng/ml) significantly higher when compared with the control group (0.12 ± 0.12 ng/ml). IL-8 and CRP had no statistically significant variation when compared to primary versus metastatic melanoma groups. Positive correlations with statistical significance were determined between anti-GM1 and CRP, respectively, IL-8, between anti-GD1a and CRP, respectively, IL-8 (Figure 3). Negative significant correlations were observed between anti-GM3, anti-GT1b, and CRP, respectively, IL-8 (Figure 3). High levels of CRP and IL-8 were associated with an increase in anti-GM1, anti-GD1a, and a decrease in anti-GM3, anti-GM2 antibodies of IgM type (Table 4).

## 4. Discussions

Melanoma, the most aggressive skin tumour is a multifactorial cancer, being the result of the interplay between genetic, immunological, and environmental factors [24–27]. Gangliosides, due to their expression on tumor cells, have been involved in tumor biology and immunogenicity and hence have been considered as targets for cancer immunotherapy [27–30]. The probability that some tumour-associated ganglioside determinants induce a human immune response generated much interest in medical research [6, 8, 11, 31–42]. If endogenously synthesized antiganglioside antibodies react only with human cancer cells, these antibodies could play an important role in the host's protective immunity to the tumor. There is little information about quantitative variations of serum antigangliosides, their origin, and progression of melanoma. Tumour ganglioside antigens generate a significantly increased immune response in patients with primary melanoma versus metastatic melanoma. In our study, the host's ability to generate an early antiganglioside response is supported by a significantly increased titter of IgM antibodies in patients with primary versus metastatic melanoma and the control group, for anti-GM1, anti-GM2, anti-GM3, anti-GD1a, anti-GD1b, anti-GT1b, and anti-GQ1b (Figure 2). The range of antiganglioside antibodies could serve as an indicator of differentiation between patients with primary melanoma and metastatic melanoma. Based on our findings, we estimate that the levels of the antiganglioside antibodies could provide information regarding the clinical staging of melanoma.

In addition, the capacity of patients to develop an antiganglioside response in the early stage of development of melanoma could be understood as a mean of defence of the body, through eliminating a danger signal from the tumour microenvironment, represented by the stimulation of glycosphingolipid synthesis [38, 40]. Synthesis of antiganglioside antibodies could confer a survival advantage in patients with primary melanoma [8]. In metastatic melanoma patients, we observed a reduction of antiganglioside antibody synthesis, a result that could suggest the immunosuppressive effect exerted by the overproduction of gangliosides associated with tumour metastasis and/or due to the overall decreased immune response.

It has been shown in a previous study that in patients with untreated primary melanoma, there is a significant statistical correlation between anti-GM1 type IgM level and clinical stage of the disease, Breslow index, Clark level, tumour localization, histologic type, presence/absence of ulceration [8, 11]. In our study, patients with cutaneous melanoma had detectable levels of anti-GM1 in primary stages. The presence of a positive significant relationship between IgM anti-GM1 level and IL-8 and CRP in our study justified our statement regarding the involvement of these antibodies in tumour proliferation by stimulating inflammation [11, 43–45]. The present study is the first one that evaluated the relation between antiganglioside antibodies and IL-8 and CRP, based on their role in melanoma diagnosis, progression, and outcome [14, 46–49], in metabolic disorders [50, 51].

Previous studies in patients with prostate cancer [35] or sarcoma [10] have shown that anti-GM1 antibodies had no diagnostic or prognostic value in these pathologies. In patients with differentiated thyroid cancer, anti-GM1 type IgG and IgM were associated with carcinogenesis, but the lack of correlation between antibody level and clinical status indicated that anti-GM1 had no diagnostic value in differentiated thyroid cancer [37].

Another study performed by our group showed that patients with primary melanoma with a high level of IgM anti-GM3 had a favourable prognosis compared with patients displaying a low antibody titer [8]. In a study on patients with primary untreated melanoma, (stages I and II), lymph nodes clear of metastasis, it was shown that the anti-GM2 antibody titer for IgM-type was not differentiated in correlation to the tumor thickness. For anti-GM3, it was obtained a direct relationship between the serum titer and the thickness of the tumor [36]. Anti-GM2 and anti-GM3 antibodies have no diagnostic significance in thyroid cancer due to the low prevalence of these antibodies [37]. In our present study, anti-GM3 negative correlations with IL-8 and CRP are suitable with the hypothesis that patients with primary melanoma with a high level of IgM anti-GM3 have a favourable prognosis.

GD1a was thought to generate an immune response in patients with early-stage melanoma [8]. In patients with T1/T2 stage prostate cancer, there were identified increased IgM anti-GD1a values compared to the T3/T4 stage, which sustains the development of an early endogenous immune response, able to eliminate the danger signal from the tumor microenvironment. These data support the role of anti-GD1a in the early diagnosis of localized prostate disease [36]. The anti-GD1a IgM-type titer was defined as a negative predictive factor of survival in patients with soft tissue sarcoma [31] and in patients with primary melanoma [8]. In patients' serum diagnosed with ovarian cancer an increased titer of IgM anti-GD1 was found, the authors pointing out that these antibodies could represent immunological markers associated with ovarian cancer progression [36]. In cutaneous melanoma, IgM anti-GD1a could be considered a marker associated with melanoma progression based on the negative correlation with CRP and IL-8 as shown in our study.

The anti-GD1b immune response in patients with gastric neoplasm can be used as a prognostic marker [36]. On the contrary, the lack of correlation between the presence of anti-GD1b and the clinical status of patients with thyroid cancer has indicated that antigangliosides do not have diagnostic significance in this neoplasm [37].

The anti-GT1b titer can be an overall positive factor associated to global survival in sarcoma [10]. Anti-GD1b, GT1b, and GQ1b antibodies that are negatively correlated with IL-8 and CRP suggest that they could indirectly suppress tumor growth and angiogenesis. Anti-GD1b, -GT1b, and -GQ1b (IgM type) influence the progression of melanoma [8, 52], soft tissue sarcomas [32, 33], Ehrlich subcutaneous solid tumors [11, 12], Ehrlich carcinomas accompanied by ascites [11, 12], and gastric cancer [34].

Our study limitations could be considered the semiquantitative assessment method of antiganglioside antibodies as a quantitative determination technique could offer more sensitive data, opening the door for further studies. One more important limitation of the study is that we evaluated the correlation between antiganglioside antibodies and inflammation markers in melanoma (IL-8 and CRP) only after or between the surgical treatment of melanoma, newer therapies being also in place. This could be the first study, which other researchers and clinicians can use and analyze in order to evaluate the influence of different melanoma treatments on antiganglioside antibodies. The pathogenic mechanisms involved in melanoma are complex [53–55]; therefore, the evaluation during the follow-up period of melanoma patients at different points is needed for a better antiganglioside profile characterization.

## 5. Conclusions

Antiganglioside antibodies anti-GM1 and -GD1a were positively correlated, while anti-GM3, -GD1b, and -GT1b were negatively associated with the inflammatory markers, IL-8, and CRP. The host's ability to elaborate an early antiganglioside response could be considered as a defence mechanism, directed toward eliminating a danger signal from the tumour microenvironment. Moreover, our results suggest that tumour ganglioside antigens generate a significantly increased immune response in patients with primary versus metastatic melanoma. Antiganglioside antibodies associated with inflammation markers could be used as diagnostic, monitoring, and treatment tools in patients with cutaneous melanoma.

## Figures and Tables

**Table 1:** Test strips coated with parallel lines of purified antigens.

Antigen	Ganglioside type	Source	Structure
GM1	Monosialoganglioside GM1	Bovine brain	Gal-3GalNAc-4[Neu5Ac-3]Gal-4GlcCer
GM2	Monosialoganglioside GM2	Bovine brain	GalNAc-4[Neu5Ac-3]Gal-4GlcCer
GM3	Monosialoganglioside GM3	Dog erythrocytes	Neu5Ac-3Gal-4GlcCer
GD1a	Disialoganglioside GD1a	Bovine brain	Neu5Ac-3Gal-3GalNAc-4[Neu5Ac-3]Gal-4GlcCer
GD1b	Disialoganglioside GD1b	Bovine brain	Gal-3GalNAc-4[Neu5Ac-8Neu5Ac-3]Gal-4GlcCer
GT1b	Trisialoganglioside GT1b	Bovine brain	Neu5Ac-3Gal-3GalNAc-4[Neu5Ac-8Neu5Ac-3]Gal-4GlcCer
GQ1b	Tetrasialoganglioside GQ1b	Bovine brain	Neu5Ac-8Neu5Ac-3Gal-3GalNAc-4[Neu5Ac-8Neu5Ac-3]Gal-4GlcCer Glc, glucose; Gal, galactose; GalNAc, N-acetyl-galactosamine

**Table 2:** IgM antiganglioside signal intensity in all groups.

Antibodies	Class	*p* significance		
		PM vs. MM	PM vs. control	MM vs. control
Anti-GM1	IgM	0.017	0.032	0.04
Anti-GM2	IgM	0.006	0.002	0.04
Anti-GM3	IgM	0.011	0.043	0.05
Anti-GD1a	IgM	0.001	0.001	0.04
Anti-GD1b	IgM	0.02	0.013	0.05
Anti-GT1b	IgM	0.016	0.011	0.05
Anti-GQ1b	IgM	0.05	0.044	0.05

MM: metastatic melanoma, PM: primary melanoma, *p*: statistical significance.

**Table 3:** IL-8 and CRP in all studied groups.

Study group	CRP (mg/dl)	*p* significance	IL-8 (pg/ml)	*p* significance
Primary melanoma	1.07 ± 0.88	0.02	68.9 ± 17.2	0.03
Metastatic melanoma	1.85 ± 0.62	0.03	74.2 ± 24.2	0.04
Control group	0.12 ± 0.12	—	10.9 ± 4.6	—

**Table 4:** Anti-GM1, -GM2, -GM3, -GD1a, -GD1b, -GT1b, and -GQ1b relation with inflammatory markers in primary melanoma group.

	CRP	IL-8
Anti-GM1	*r* = 0.33 *p* ≤ 0.001	*r* = 0.37 *p* ≤ 0.001
Anti-GM2	*r* = −0.06 *p* = 0.47	*r* = −0.03 *p* = 0.67
Anti-GM3	*r* = −0.17 *p* = 0.05	*r* = −0.15 *p* = 0.02
Anti-GD1a	*r* = 0.45 *p* ≤ 0.001	*r* = 0.53 *p* ≤ 0.001
Anti-GD1b	*r* = −0.24 *p* ≤ 0.001	*r* = −0.22 *p* ≤ 0.001
Anti-GT1b	*r* = −0.29 *p* ≤ 0.001	*r* = −0.25 *p* ≤ 0.001
Anti-GQ1b	*r* = −0.13 *p* = 0.11	*r* = −0.15 *p* = 0.09

## Data Availability

The data used to support the findings of this study are included within the article.
